# Supplementary material for: The Potential of Ectomycorrhizal Fungi to Modulate below and Aboveground Communities May Be Mediated by 1-Octen-3-ol
Source: J Fungi (Basel). 2023 Jan 29;9(2):180. doi: 10.3390/jof9020180 (PMC9961352; doi:10.3390/jof9020180)
Supplement: Supplementary file 1 [file jof-09-00180-s001.zip › jof-2147757-supplementary.pdf]

Table S1. Effects of 1-octen-3-ol 1 μM (VOC) and ECM mycelium on Cistaceae germination rates (%, n=5). Treatments: **Tlep** - *Terfezia leptoderma*; **Ldel** - *Lactarius deliciosus*; **VOC** - 1-octen-3-ol 1 μM; **CT** – control. Data were compared using a Kruskall–Wallis test. Post hoc comparisons were made using a Dunn's test, respectively. Data are means and standard deviation (SD). Different letters indicate significance at $p < 0.05$, of each Cistaceae species in each treatment.

	Cistus albidus			*Cistus ladanifer*			*Cistus psilosepalus*		
**Treatment**	**Mean**	**SD**		**Mean**	**SD**		**Mean**	**SD**	
**CT**	30.00	± 11.18	a,b	50.00	± 17.68	a	35.00	± 13.69	a,b
**Tlep**	35.00	± 13.69	a,b	50.00	± 17.68	a	30.00	± 11.18	a,b
**Ldel**	50.00	± 17.68	a	35.00	± 13.69	a	65.00	± 13.69	a
**VOC**	20.00	± 11.18	b	60.00	± 13.69	a	20.00	± 11.18	b

	Cistus salviifolius			*Halimium halimifolium*			*Tuberaria guttata*		
**Treatment**	**Mean**	**SD**		**Mean**	SD		**Mean**	**SD**	
**CT**	40.00	± 13.69	a	50.00	± 17.68	a,b	40.00	± 22.36	b
**Tlep**	40.00	± 13.69	a	65.00	± 13.69	a	55.00	± 32.60	a,b
**Ldel**	50.00	± 17.68	a	35.00	± 13.69	a,b	95.00	± 11.18	a
**VOC**	30.00	± 11.18	a	30.00	± 11.18	b	85.00	± 22.36	a,b
